# Opportunities for Success

**Published:** 1899-05

**Authors:** J. H. Grant

**Affiliations:** Palestine, Texas.


ARTICLE XI.

### OPPORTUNITIES FOR SUCCESS.

J. H. GRANT, D. D. S., PALESTINE, TEXAS.

It has been truly said that "The world pushes a professional man the way his intelligent accomplishments and inclinations wish him to go." If he is ambitious and wants to go up it pushes him up—if going down, it pushes him down, gravitation; however, making the speed greater on the decline.

A successful life has its moments of strength and bloom, its bright moments of inspiration, in which the human artist, the painter of human life, seizes on and utters what is purest, most beautiful and divine. If, in our human life, we acted only then, if then all sacrifices were made, all victories won, there would be but little difficulty in making not only our human life but our professional life a complete success. When at our best we but fill our alloted space as a special mission. It might be said that each of us in our professional sphere is a bottle on the shelf of time on which is placed a special label, for a particular position, and by it we steadily and regularly, even though sometimes struggling against it ourselves, or though struggled against by others, either fall or stand in our appropriate places. Men of real merit will, if they persevere, at last reach the station to which they are entitled, and pretenders, intruders and quacks will be ejected with contempt and derision. I feel that it is no small evil that the avenues to professional fame should be blocked up by a swarm of noisy, pushing, elbowing set of pretenders with no other accomplishments than cheek and a mania for outdoor advertising, and who cannot make good their entrance, but sadly hinder in the meantime those who have a right to enter. Those who have pride and who, by their application and hard study, acquire accomplishments and who will not disgrace themselves by joining in the unseemly scuffle, must expect to feel at first as if hustled and shouldered back, while some men of talents, accordingly, turn away in dejection from pursuits in which success appears to bear no proportion to deserts. But there are some who have sufficient confidence in their own powers and sufficient elevation of mind to wait with serene and contemptous patience, while quack after quack presses before them.

It is the conscientious man that struggles most at the beginning of his professional career, but will have less anxiety on the home stretch. Environments often place men in hard luck, but never fear disadvantages; a clear conscience, backed up by good discipline and a determined persistency, will overcome these appearing obstacles and clear the way to honor and respect (though seemingly) over a rough way.

Be a man, mind your own business and you will forge ahead and then your attention will be less attracted by the side-shows that often perform in your town, and gradually competition will be less—your field will be clear, your position and reputation well established and your success assured.—*Texas Dental Journal.*

